# Sensory Integration Regulating Male Courtship Behavior in *Drosophila*

**DOI:** 10.1371/journal.pone.0004457

**Published:** 2009-02-13

**Authors:** Dimitrije Krstic, Werner Boll, Markus Noll

**Affiliations:** 1 Institute for Molecular Biology, University of Zürich, Zürich, Switzerland; 2 Ph.D. Program in Molecular Life Sciences, Zürich, Switzerland; Freie Universitaet Berlin, Germany

## Abstract

The courtship behavior of *Drosophila melanogaster* serves as an excellent model system to study how complex innate behaviors are controlled by the nervous system. To understand how the underlying neural network controls this behavior, it is not sufficient to unravel its architecture, but also crucial to decipher its logic. By systematic analysis of how variations in sensory inputs alter the courtship behavior of a naïve male in the single-choice courtship paradigm, we derive a model describing the logic of the network that integrates the various sensory stimuli and elicits this complex innate behavior. This approach and the model derived from it distinguish (i) between initiation and maintenance of courtship, (ii) between courtship in daylight and in the dark, where the male uses a scanning strategy to retrieve the decamping female, and (iii) between courtship towards receptive virgin females and mature males. The last distinction demonstrates that sexual orientation of the courting male, in the absence of discriminatory visual cues, depends on the integration of gustatory and behavioral feedback inputs, but not on olfactory signals from the courted animal. The model will complement studies on the connectivity and intrinsic properties of the neurons forming the circuitry that regulates male courtship behavior.

## Introduction

An important objective of behavioral biology is to understand how the brain integrates external stimuli and evokes an appropriate response [1], [2]. Courtship is one of the most robust and sophisticated behaviors, as sexual reproduction crucially depends on it. It has been extensively characterized in *Drosophila* [3]–[7] and used to study how the brain regulates a largely innate complex behavior that depends on multiple sensory inputs [5], [6], [8]. A male perceives a potential mate through his visual, olfactory, and gustatory senses that direct him to initiate courtship [3], [6], [9], which in turn elicits a response from the courted female or male [10], [11]. The signals emitted by the courted fly provide the courting male with information about gender, conspecificity, receptivity, and sexual fitness. These signals are then converted within the male into a response, which manifests itself in the various steps of his courtship behavior [3]–[6].

The decisive question of how the male brain transforms the sensory inputs into an innate behavioral response addresses two entirely different aspects: (i) what is the structure of the neural circuit performing this task, the ‘hardware’, and (ii) what is the program that controls this behavior, the ‘software’. While many laboratories have investigated the architecture of the neural circuit regulating courtship behavior [12]–[16], we aim here at elucidating the logic of its program [17], [18], following the rationale that the male's courtship behavior, the output, is related to the sensory input through the program executed by the underlying neural network. This approach requires a systematic analysis of the influence on male courtship behavior of the various sensory inputs. While a wealth of results describes the impact of gustatory [19]–[24], olfactory [16], [25]–[28] and visual [29]–[32] cues on male courtship behavior, few studies have examined the impact of their integration on courtship [17], [33], [34].

To determine how the male integrates different sensory signals during courtship, we used combinations of mutations, transgenes, and ablations that eliminate single sensory modalities in the male but do not affect the processing functions of the central nervous system. From the resulting changes in male courtship behavior we have derived a model describing the logic of the program that integrates the sensory information important for courtship behavior and sexual orientation of the *Drosophila melanogaster* male in single-choice courtship assays. This model thus illustrates the logic of the underlying neural network regulating this complex innate behavior.

## Results

### Experimental approach

Our analysis is based on single-choice courtship assays, in which a sexually mature male is offered a wild-type receptive virgin female or mature male. Courtship was observed in a mating chamber whose dimensions do not seriously restrict behavioral display (see Materials and Methods). To distinguish between initiation and maintenance of courtship, the performance of the male was measured by three parameters: (i) the fraction of males initiating courtship by extending and vibrating a wing (love song), (ii) the latency till courtship initiation, and (iii) the courtship vigor index, cvi, defined as the fraction of time the male spent courting from courtship initiation until copulation or the end of observation at 10 minutes. The average latency and cvi are computed by taking into account only the fraction of males that initiated courtship. Though less informative than the combination of these three parameters, the courtship index CI, usually used as single parameter to describe the intensity of male courtship and defined as the fraction of the observation period during which any courtship behavior occurs [35], has been computed for each experiment as well (Figure S1).

**Figure 1:**
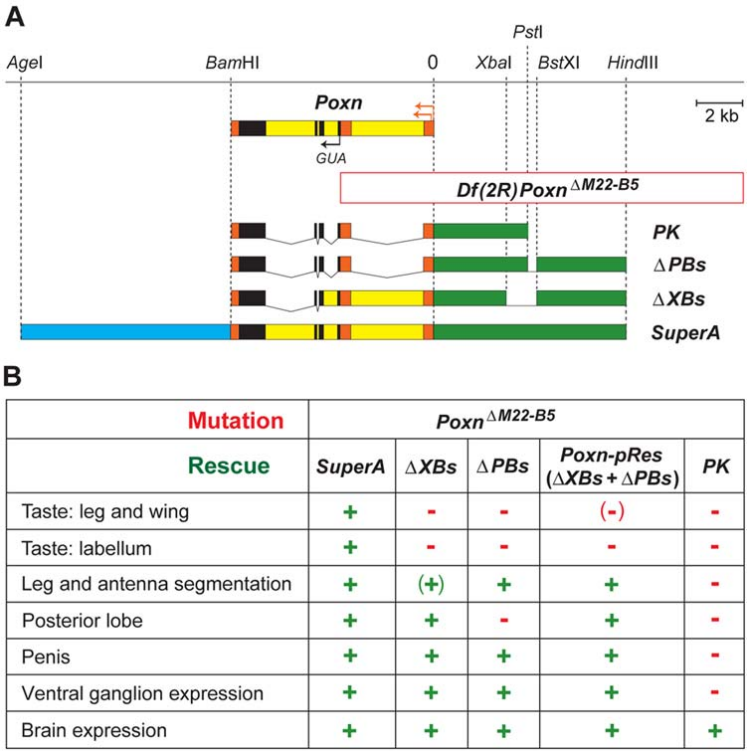
Poxn null allele and rescue transgenes used for manipulation of gustatory modality. (A) Map of the *Poxn* gene [41], [42], the *Poxn^ΔM22-B5^* deletion [42], and the *Poxn* rescue constructs. The *Poxn* transgenes *ΔPBs*, *ΔXBs*, and *SuperA*, and the *Poxn* deficiency *Df(2R)Poxn^ΔM22-B5^* are shown with regard to a restriction map of the *Poxn* locus. Upstream region (green), 5′ leader and 3′ trailer (orange), coding region (black), introns (yellow), and downstream region (blue) are indicated. (B) Table of *Poxn* transgenes used to rescue development of antenna, leg, male genitalia, CNS, and of all or only very few gustatory bristles in *Poxn^ΔM22-B5^* null mutants. The *Poxn-SuperA* transgene (*SuperA*) rescues all mutant phenotypes of the *Poxn* gene [42]. Since the *ΔXBs* transgene does not completely rescue the leg/antenna segmentation phenotype of *Poxn^ΔM22-B5^* null mutants, it was combined with one copy of the *ΔPBs* transgene. This combination, *Poxn-pRes*, rescued leg and antennal segmentation but also, in a random manner (data not shown), 2–4 of ∼50 taste bristles on the foreleg of a wild-type male. The genotypes *Poxn-pRes* and *Poxn-SuperA* are short for *ΔXBs6*; *Poxn^ΔM22-B5^*/*Poxn^ΔM22-B5^ ΔPBs96.2* and *Poxn^ΔM22-B5^ SuperA-158*, respectively. The *SuperA* transgene also rescued all courtship mutant phenotypes of *Poxn-pRes* males described in this paper (data not shown), when combined with the *ΔXBs* and *ΔPBs* transgenes (*ΔXBs6*; *Poxn^ΔM22-B5^ SuperA-158*; *ΔPBs69*/+). This demonstrates that the insertions of the *ΔXBs* and *ΔPBs* transgenes do not interfere with the rescue of the *Poxn* mutant phenotype by the *SuperA* transgene. *PK6* is a *Poxn* transgene that does not rescue any taste bristles [42]. *Poxn-PK6*; *Or83b^2^* males showed no initiation of courtship in the dark (data not shown). However, we did not use these flies in our courtship assays because they lack the *Poxn* functions required for proper development of male genitalia as well as the *Poxn* ventral ganglion function, which all may not influence courtship initiation but interfere with copulation [42].

To discriminate between effects on male courtship behavior through visual, olfactory, and gustatory stimuli, we eliminated their perception in the courting male by mutations that affect single senses but not the processing functions of the central nervous system. Thus, the *Or83b^2^* mutant allele [36] of the broadly expressed olfactory receptor gene *Or83b* [36]–[38] was used to interfere with olfaction. Since a functional *Or83b* product is essential for the proper localization and function of co-expressed olfactory receptors [39], [40], the olfactory response of *Or83b^2^* mutant flies is strongly reduced [36]. Gustatory perception was abolished in a *Pox neuro* (*Poxn*) [41] null mutant, *Poxn^ΔM22-B5^*, whose taste bristles are transformed into mechanosensory bristles [42]. To these males all *Poxn* functions important for courtship, except those required for taste bristle development, were supplied by two *Poxn* transgenes (*Poxn-pRes*; Figure 1), while in control males all *Poxn* functions were rescued by a complete *Poxn* transgene (*Poxn-SuperA*; Figure 1) [42]. Finally, we tested the role of vision by observing courtship under dim red light (dark), which to flies is darkness [30]. Alternatively, the courting males were blinded by black paint covering their eyes or by the *ninaB^360d^* mutation [43], which blocks the synthesis of the rhodopsin chromophore retinal [44], [45].

As the courted animal reacts and adapts to the courting male, it emits a multitude of behavioral cues [10], [11]. These may consist of auditory, visual, and/or mechanosensory signals, which are induced by the approaching courter male and may have a positive or negative impact on his courtship behavior. To assess the importance of these behavioral signals, in the following collectively termed ‘feedback behavior’, they were abolished by decapitation of the courted animal.

### Scanning courtship strategy in the dark

**Figure 2:**
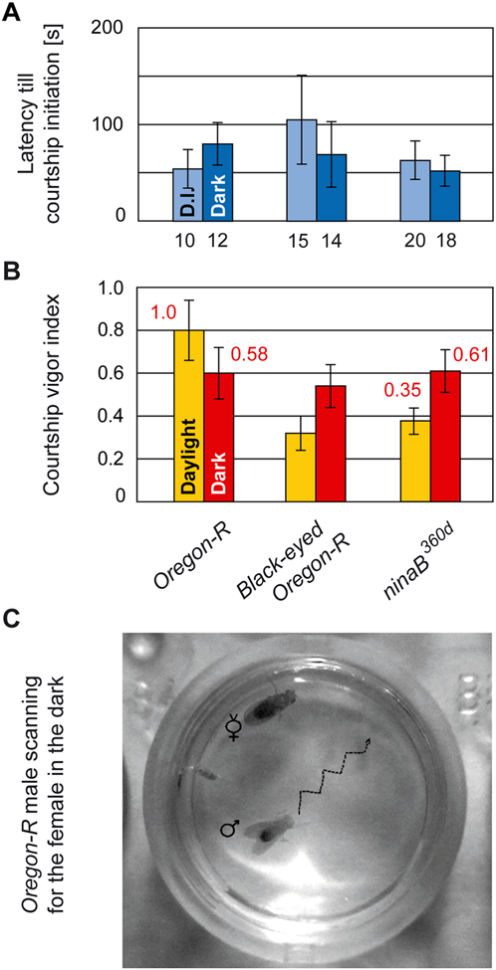
Light-dependent adaptation of male courtship strategy: visual tracking versus scanning. (A) Average latency (in seconds) till courtship initiation, and (B) courtship vigor index were measured in single-choice courtship assays with mature males of indicated genotypes and receptive *Ore-R* virgin females in daylight (light colored columns) or under dim red light (dark colored columns). The fraction of males initiating courtship was 100% in all cases. In this and all other figures, the numbers below columns indicate the number of couples observed, unless indicated differently, and error bars always represent double standard errors of the mean. Red numbers above columns (B) denote copulation efficiencies. The copulation efficiencies of black-eyed *Ore-R* males are considerably reduced and hence not indicated. (C) Photograph of male (♂) in search of virgin (V) under infrared light. The dotted line indicates the zigzag course of the male scanning the mating chamber.

Although *Drosophila melanogaster* males preferably court in the dark [46], they also court during daylight in early mornings and late afternoons [3], which coincide with their active periods at dawn, dusk [47], and night [48]. To investigate the influence of light on courtship, we observed wild-type *Oregon-R* (*Ore-R*) males courting receptive virgin females in single-choice courtship assays in daylight and darkness. All *Ore-R* males initiated courtship independently of light with a short latency (Figure 2A), while their cvi was only slightly reduced in the dark compared to daylight (Figure 2B; p = 0.044). This high cvi in the dark results from a remarkable change in male courtship behavior. When a female decamps in response to a courting male in daylight, the male pursues her by visual tracking (M1; henceforth M followed by a number refers to the number shown in the model presented in the Discussion). However, when a female decamps in the dark, the male searches her by spreading his wings and scanning the mating chamber in a zigzag course (M2; Figure 2C, Movie S1). Males left alone in a chamber do not scan, either in the dark or in the light or under a ‘feminine sky’, i.e., in a chamber preconditioned by the presence of receptive virgin females [25] (data not shown).

It seemed plausible that scanning, triggered by the decamping female in the dark, is supported by olfactory cues, as other animals have been reported to move in a zigzag pattern when following olfactory gradients [49], [50]. However, *Drosophila* males with either gustatory (*Poxn-pRes*) (M3) or olfactory (*Or83b^2^*) deficits (M4) displayed the scanning behavior (data not shown), which suggests that volatile or contact pheromones are sufficient to drive this behavior in the dark.

Males impaired for visual tracking by the *ninaB^360d^* mutation or black paint covering their eyes all initiated courtship and as efficiently as *Ore-R* males, both in daylight and in the dark (Figure 2A). However, in daylight these males did not compensate for their blindness by switching to the scanning behavior (Movie S2) and displayed a reduced cvi compared to that of *Ore-R* males (Figure 2B; p<0.001 for both cases). By contrast, in the absence of light, these males applied the scanning strategy and maintained a courtship vigor comparable to that of wild-type males (Figure 2B). This suggests that lack of visual acuity is not sufficient for blinded males (black-eyed or *ninaB^360d^*) to adopt the scanning behavior in daylight for the pursuit of a decamping female, but that it is the perception of light that controls this behavior (M5).

In summary, these results show that visual cues, though dispensable for courtship initiation, are necessary for the maintenance of high courtship intensity in daylight. In the dark, the neuronal network of the male is functionally modified to be independent of visual input and to rely on a scanning strategy. This strategy represents an effective way to restore contact with the decamping female and hence to enhance the courtship vigor and thus the copulation efficiency in the dark (*ninaB^360d^* in Figure 2B; p = 0.03; for definition of copulation efficiency, see Materials and Methods).

### Interplay of senses during heterosexual courtship

**Figure 3 pone-0004457-g003:**
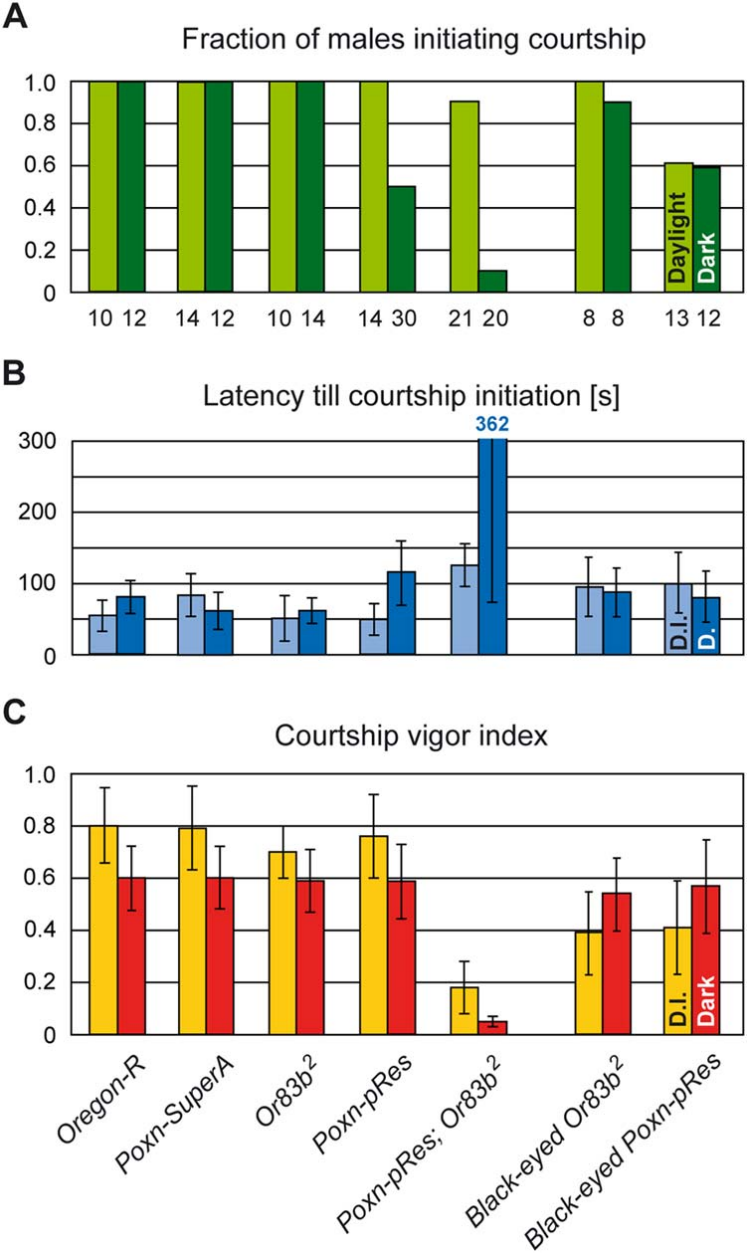
Chemosensory signals drive male courtship in the presence and absence of light. Male courtship parameters, (A) the fraction of males initiating courtship, (B) the average latency (in seconds) till courtship initiation, and (C) the courtship vigor index, were measured in single-choice courtship assays with mature males of indicated genotypes and intact receptive *Ore-R* virgins in daylight (light colored columns) or under dim red light (dark colored columns).

Males with strongly reduced chemosensation displayed virtually no courtship activities in the dark (*Poxn-pRes*; *Or83b^2^* in Figure 3). Only 10% of these males initiated courtship (Figure 3A) with a long latency (Figure 3B), and their courtship was limited to a few seconds of wing extension (Figure 3C). Since males, when left alone in the dark, do not show any courtship behavior, we attribute this marginal courtship activity of *Poxn-pRes*; *Or83b^2^* males in the presence of a female to the fact that 2–4 of about 50 taste bristles per male foreleg have been rescued [42], [51] (see also legend to Figure 1). This experiment confirmed the high efficiency by which the pheromonal chemosensation is reduced in *Poxn-pRes* and *Or83b^2^* males. Similarly, *Poxn-pRes* males whose olfaction was eliminated by the removal of the antennae and maxillary palps did not initiate courtship towards females either (data not shown). However, as this surgical manipulation had side effects on courtship maintenance in the dark, we did not use such males for further analysis.

When males were deprived of gustatory but not olfactory senses, only half of them initiated courtship in the dark (*Poxn-pRes* in Figure 3A), but their latency did not differ significantly from those of wild-type *Ore-R* and *Poxn-SuperA* control males (Figure 3B; p = 0.13 and p = 0.055). After courtship initiation, *Poxn-pRes* males courted females with the same vigor as did wild-type or control males (Figure 3C). These results indicate that the olfactory sense alone, though not very efficient for initiation (M6), is able to maintain a high courtship intensity in the dark (M3). By contrast, the impairment of the male's olfaction by the *Or83b^2^* mutation did not affect his courtship performance in the dark, as compared to *Ore-R* males (Figure 3), which suggests that the gustatory sense is sufficient to trigger courtship (M7) and elicit a high courtship vigor (M4).

In daylight, flies impaired for both olfactory and gustatory perception (*Poxn-pRes*; *Or83b^2^*) and thus relying only on visual cues, initiated courtship fairly reliably (Figure 3A) and rather quickly (M8) (Figure 3B; p = 0.042 and p<0.001 compared to *Poxn-SuperA* and *Ore-R* controls). However, they revealed a strongly reduced cvi (Figure 3C; p<0.001 compared to *Poxn-SuperA*) because the male does not track the decamping female (M9). By contrast, males whose vision was supplemented with either olfactory (*Poxn-pRes*) or gustatory senses (*Or83b^2^*) courted with the same intensity as wild type (M10) (Figure 3C). These results suggest that visual cues, though sufficient to trigger courtship, are inefficient in maintaining a high courtship vigor without the support of gustatory or olfactory cues.

Flies impaired for vision and gustation (black-eyed *Poxn-pRes*) or vision and olfaction (black-eyed *Or83b^2^*) showed no difference in courtship initiation between daylight and darkness (Figure 3A,B), which demonstrates that the importance of single chemosensory modalities for courtship initiation is not changed in the presence or absence of light (M6, M7). However, single chemosensory modalities drive courtship in daylight less efficiently in the absence than in the presence of vision (cf. *Poxn-pRes* and *Or83b^2^* with black-eyed *Poxn-pRes* and black-eyed *Or83b^2^* in Figure 3C; p = 0.01 and p = 0.005, respectively) because a blind male is unable to track a decamping female (M11, M12).

### Sexual orientation: decisive gustatory and behavioral signals

**Figure 4 pone-0004457-g004:**
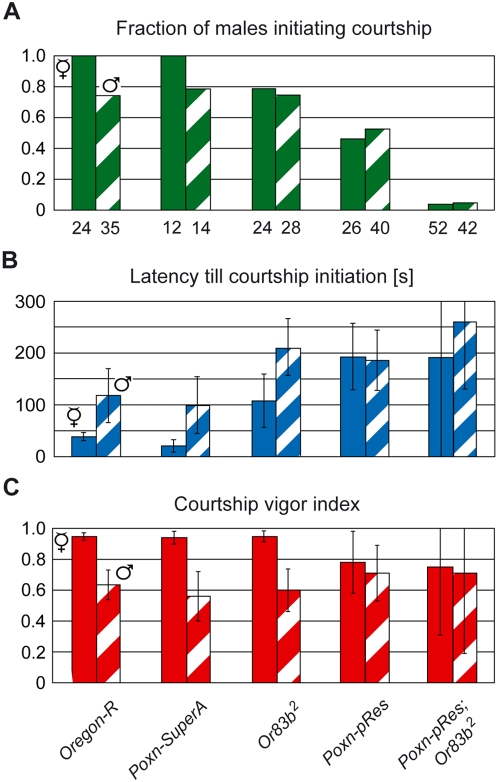
Gustatory, but not olfactory, signals of the courted fly contribute to the heterosexual orientation of the courting male. (A) The fraction of males initiating courtship, (B) the average latency (in seconds) till courtship initiation, and (C) the courtship vigor index were measured in single-choice courtship assays, performed under dim red light with courting males of indicated genotypes and decapitated receptive *Ore-R* virgins (V, filled columns) or decapitated mature *Ore-R* males (♂, hatched columns).

To assess the importance of the different senses for the sexual orientation of males, we first observed males in single-choice courtship assays in the dark with object animals that had been decapitated to prevent their feedback behavior. All *Ore-R* and *Poxn-SuperA* control males initiated courtship towards decapitated females (Figure 4A) with a short latency (Figure 4B) and courted them vigorously until the end of observation (Figure 4C). Copulation with decapitated females was never observed even though males bent their abdomen and attempted to copulate, which suggests that positive feedback from the female is crucial for copulation (M13). When confronted with decapitated males, the fraction of *Ore-R* and *Poxn-SuperA* males initiating courtship was somewhat reduced (Figure 4A; p = 0.008 and p = 0.1, respectively, for comparison of courtship towards decapitated females and males), and the courtship latency significantly increased (Figure 4B; p = 0.006 and p = 0.04, respectively). *Ore-R* and *Poxn-SuperA* males also courted decapitated males intensely, yet with a cvi significantly reduced compared to that towards decapitated females (Figure 4C; p<0.001 for both cases).

When olfaction was impaired by the *Or83b^2^* mutation, a significantly reduced fraction of *Or83b^2^* males, as compared to *Ore-R* males, initiated courtship towards decapitated females in the dark (M14) (Figure 4A; p = 0.02) and equaled the fraction of *Or83b^2^* males initiating courtship towards decapitated males (M15) (Figure 4A, p = 0.80). The latency of these *Or83b^2^* males till courtship initiation towards decapitated females was prolonged compared to that of *Ore-R* males (Figure 4B, p = 0.008), but was still significantly shorter than that towards decapitated males (Figure 4B, p = 0.03). *Or83b^2^* males courted decapitated females (M16) and males (M17) vigorously, but were able to discriminate between the two sexes as effectively as *Ore-R* controls (Figure 4C; p<0.001). By contrast, males lacking taste bristles displayed no preference for courtship initiation towards decapitated females (M18) or males (M19) (*Poxn-pRes* in Figure 4A,B; p = 0.5 in A, p = 0.9 in B), with only half of them initiating courtship (*Poxn-pRes* in Figure 4A). Moreover, these males courted decapitated females (M20) and males (M21) with indistinguishable high cvis (*Poxn-pRes* in Figure 4C; p = 0.61). These results imply that gustatory, but not olfactory, cues carry information on the sex of the courtee. However, it should be emphasized that gustatory signals, in the absence of feedback and visual cues, do not inhibit male–male courtship, as males with intact gustation and olfaction (*Poxn-SuperA*), and males whose gustation was eliminated (*Poxn-pRes*) courted decapitated males with the same high cvi (Figure 4C; p = 0.19). It rather seems that the severe reduction of the gustatory perception diminishes the female attractiveness in the courting male (see also Figure S1B), whereas his olfactory sense receives equally attractive stimuli from decapitated females and males (*Poxn-SuperA* and *Poxn-pRes* in Figure 4A–C).

In the absence of both chemical modalities (*Poxn-pRes*; *Or83b^2^*), the fraction of males initiating courtship was marginal (Figure 4A), but these males courted decapitated flies vigorously (Figure 4C). This result differs drastically from that obtained with intact females (*Poxn-pRes*; *Or83b^2^* in Figure 3C) because decapitated animals are immobile and unable to decamp and hence induce in these males a low but constant positive stimulus sustaining their courtship vigor. Taken together, these results suggest that males receive attractive chemosensory stimuli from decapitated females as well as males. However, while olfactory stimuli of both sexes appear to be equally attractive to the male, gustatory stimuli carry sex-specific information and induce in males a clear preference for heterosexual courtship.

**Figure 5 pone-0004457-g005:**
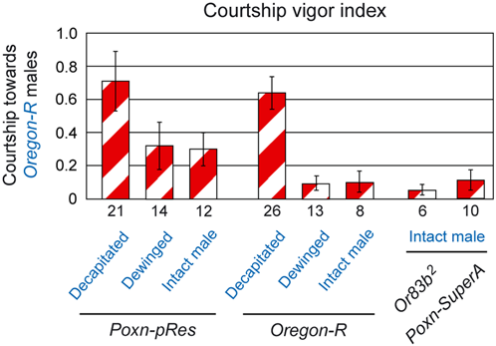
Integration of gustatory signals and feedback behavior of the courted fly enforce heterosexual orientation of males in the dark. The courtship vigor index was measured in single-choice courtship assays performed under dim red light with courting males of indicated genotypes and decapitated, dewinged, or intact males. Below each column, the number of males that initiated courtship is shown. Measurements of cvi towards decapitated males were taken from Figure 4C.

To evaluate how the feedback behavior of the courted male influences the courting male, we compared courtship in the dark towards intact males or males whose wings had been removed with that towards decapitated males. When facing a dewinged or intact male able to respond through prohibitory behavioral signals, taste-deficient males displayed a significantly reduced cvi (M22) (*Poxn-pRes* in Figure 5; p<0.001 for comparison of intact or dewinged with decapitated males). However, the cvi of these taste-deficient males was still significantly higher than that of wild-type and control males facing intact males (*Ore-R* and *Poxn-SuperA* in Figure 5; p = 0.004 and p = 0.005; see Movie S3). Occasionally, *Poxn-pRes* males were scanning the chamber when losing track of the dewinged object male (M23) in a fashion similar to that observed for heterosexual courtship in the dark (second part of Movie S3). Interestingly, scanning was not observed when object males were intact, which suggests that their wing scissoring, though not affecting the courtship vigor of taste-deficient males (p = 0.86 for comparison of cvi of *Poxn-pRes* males towards intact and dewinged males), is sufficient to inhibit their scanning (M24). In contrast to taste-deficient males, olfactory-deficient *Or83b^2^* males courted intact males with as low a cvi, as did *Ore-R* males (Figure 5). Therefore, the ablation of the olfactory sense does not compromise the sexual orientation of males.

In summary, these experiments demonstrate that the behavioral cues of the courted male act as repellents and cooperate with sex-specific gustatory signals to enforce heterosexual orientation of the male in the dark (M25), whereas olfactory signals do not permit the courting male to discriminate between males and females and provide exclusively attractive stimuli.

### Sexual orientation directed by feedback, vision, and taste

**Figure 6 pone-0004457-g006:**
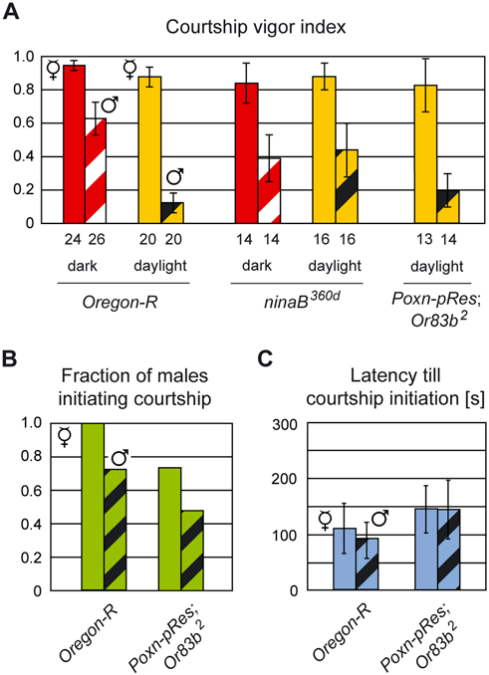
Vision strongly supports the heterosexual orientation of males. (A) Courtship vigor indices were measured in single-choice courtship assays, performed in the dark or in daylight with courting males of indicated genotypes and decapitated *Ore-R* virgins (V, filled columns) or decapitated *Ore-R* males (♂, hatched columns). The number of males that initiated courtship is shown below each column. (B) Fractions of males initiating courtship and (C) average latencies (in seconds) till courtship initiation correspond to the courtship assays in (A) of *Ore-R* and *Poxn-pRes*; *Or83b^2^* males courting decapitated flies in daylight.

In daylight, as in the dark, *Ore-R* males courted decapitated females very vigorously (Figure 6A). However, when facing a decapitated male in daylight, *Ore-R* males exhibited a cvi that was substantially reduced compared to that observed in the dark (Figure 6A; p<0.001), which suggested that also visual cues determine the sexual orientation of males. In view of the sexually dimorphic body patterns and sizes of males and females, this conclusion seemed plausible. It was corroborated by the observation that blind *ninaB^306d^* males courted decapitated males in daylight with the same cvi as in the dark (M26) (Figure 6A; p = 0.64). The results with *ninaB^360d^* males were further supported by courtship assays in daylight with males that could perceive neither gustatory nor olfactory signals. These males could clearly discriminate between decapitated males and females (*Poxn-pRes*; *Or83b^2^* in Figure 6A; p<0.001), which demonstrates that vision alone is sufficient to enforce heterosexual orientation in single-choice courtship assays (M26). Interestingly, a comparison of courtship initiation towards decapitated males and females in daylight shows that vision does not efficiently prevent *Ore-R* males from initiating courtship towards decapitated males (Figure 6B,C). Similarly, vision alone stimulates courtship initiation towards both decapitated females (M27) and males (M28) at first (*Poxn-pRes*; *Or83b^2^* in Figure 6B,C), and it is only after courtship initiation that vision reveals its strong discriminatory property (M26, M29) (*Poxn-pRes*; *Or83b^2^* in Figure 6A).

**Figure 7:**
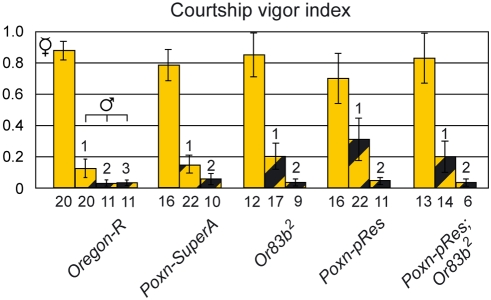
Integration of feedback with visual signals inhibits homosexual orientation of males. Courtship vigor indices were measured in single-choice courtship assays in daylight, with courting males of indicated genotypes and decapitated receptive *Ore-R* virgins (V, filled columns) or *Ore-R* males that were either decapitated (1), dewinged (2), or intact (3) (♂, hatched columns). The number of males that initiated courtship is shown below each column. Values of cvi of *Ore-R* and *Poxn-pRes*; *Or83b^2^* males courting decapitated flies in daylight were taken from Figure 6A.

Since vision plays a prominent role in promoting heterosexual courtship, both *Poxn-pRes* and *Or83b^2^* males were clearly able to distinguish between decapitated males and females in daylight (Figure 7; p<0.001 in both cases). However, in the absence of gustation, males slightly increased their courtship vigor towards decapitated males (Figure 7; p = 0.028 for comparison of *Poxn-pRes* with *Poxn-SuperA*), which indicates that gustatory together with visual cues contribute to the suppression of male–male courtship (M30).

To measure the impact of feedback behavior on male–male courtship in daylight, *Ore-R* males were assayed with dewinged and intact object males (Figure 7). These wild-type males courted intact and dewinged males with the same marginal cvi, as already observed in the dark (Figure 5). This observation suggests that behavioral cues other than wing scissoring (e.g., kicking) play an important role in preventing males from courting other males. Furthermore, for all genotypes tested, the cvi towards dewinged as compared to decapitated males was significantly reduced (Figure 7; p<0.01 in all cases), and as low as that of *Ore-R* or *Poxn-SuperA* males courting dewinged males (Figure 7). These results show (i) that in single-choice courtship assays, the integration of visual and feedback cues is sufficient to fully suppress male–male courtship, and (ii) that this integration does not depend on the presence of gustatory signals (M31).

### Chaining behavior

**Figure 8:**
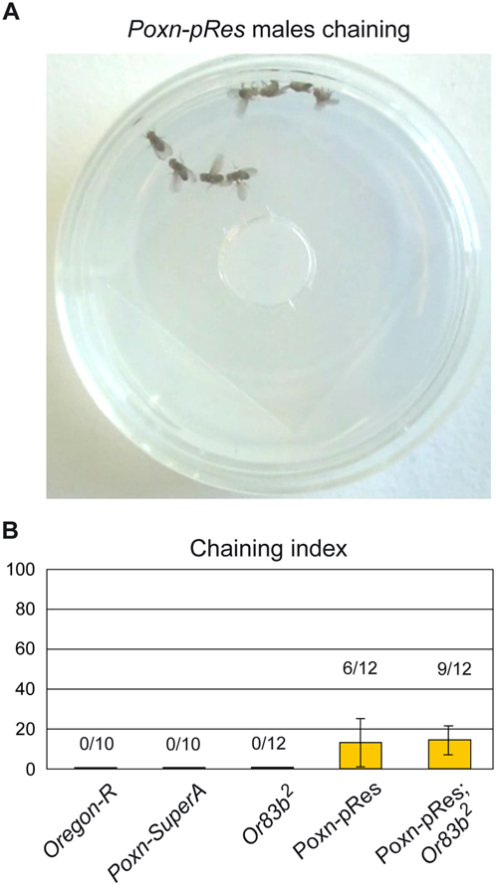
Chaining behavior of taste-deficient males. (A) Chain of four courting *Poxn-pRes* males. The picture was taken 5 minutes after eight mature, but sexually naïve, *Poxn-pRes* males were placed together into a small Petri dish. (B) In addition to the average chaining indices for groups of eight males of indicated genotypes, the number of groups for which chaining was observed over the total number of groups examined is shown for each genotype.

The chaining behavior of males [4] has also been used as a criterion to measure the intensity of male–male courtship [13]. When groups of eight *Poxn-pRes*; *Or83b^2^* males were placed in a Petri dish, some of them started courting each other and formed courtship chains (Movie S4). Courtship chains were also observed with males lacking only gustatory perception (*Poxn-pRes* in Figure 8A), but not with *Or83b^2^* males deficient only for olfactory perception or with wild-type and *Poxn-SuperA* control males (Figure 8B). In the absence of taste perception, males were forming chains of usually 3–4 individuals within an average of 5 minutes after being placed into the dish. However, chains were not observed in all groups during the observation period, and the chaining behavior, measured by the chaining index, was not very intense (*Poxn-pRes* and *Poxn-pRes*; *Or83b^2^* in Figure 8B).

## Discussion

### A model of sensory input integration in the male brain during courtship

**Figure 9:**
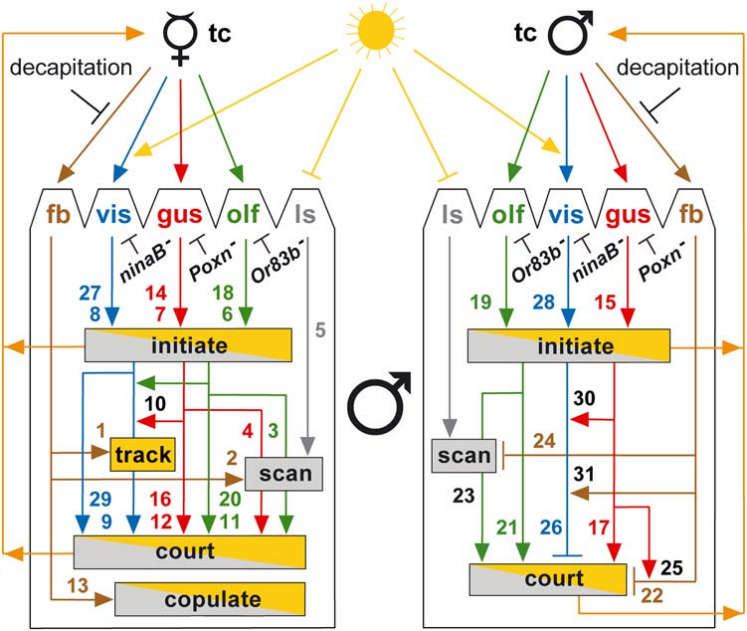
Model illustrating the regulation of *Drosophila* male courtship behavior through integration of sensory signals. The model, derived from the results presented here, illustrates how courtship activity of a male is regulated by the various sensory inputs when the male (♂) faces a receptive virgin (V), shown on the left, or another male (♂), shown on the right. The sensory receptors of the male are responding to the presence (yellow sun) or absence of light through an unidentified light sensor (ls), and to olfactory (olf), gustatory (gust), visual (vis), or behavioral feedback (fb) signals. It is unclear which senses are stimulated by the fb signals. Tactile cues (tc) that are necessary but not sufficient to stimulate male courtship originate from the object animal and are always present in our courtship assays (see Discussion). Arrows and T-bars are stimulatory and inhibitory signals affecting a modality or behavior, but do not indicate differences in relative weights. However, qualitative information on relative weights where known is provided in the text. Red, green, and blue lines relay gustatory, olfactory, and visual information, respectively. Gray lines transport signals from the light sensor, while brown lines transmit the behavioral feedback of the object animal in response to being approached and courted (orange lines). Arrows converging on the same behavioral step, illustrated by a box (yellow: daylight; gray: dark; gray/yellow: daylight or dark), may be sufficient or necessary to trigger that behavioral step. Lines passing behind the boxes for tracking and scanning behavior indicate that the corresponding inputs maintain but do not trigger these behavioral steps. Numbers refer to those in parentheses behind the experimental evidence mentioned in the text, while their color refers to that of the corresponding sensory modality. Black numbers indicate where one modality is used to stimulate another. In cases where two colored numbers refer to the same arrow, the lower number refers to assays with intact, the other with decapitated females.

Based on the behavioral studies presented here, we propose a model for the integration of the various sensory inputs that drive the courtship behavior and sexual orientation of the *Drosophila melanogaster* male (Figure 9). In the following we discuss the salient features of this model.

Clearly, olfactory (M6) and gustatory (M7) stimuli suffice to trigger males to court receptive females reliably in daylight. However, once the female decamps (M1), the male also depends on visual cues (M10) to maintain courtship efficiently. By contrast, vision alone, though sufficient to trigger courtship (M8), is not efficient in driving it (M9), but sustains the male's sex drive well if supported by the olfactory or gustatory sense (M10). These observations show that although chemosensory or visual inputs are capable of inducing and, to some extent, driving courtship (M9, M11, M12), they are not functionally redundant since their integration (M10) is necessary for vigorous and efficient courtship. To retrieve a female in the dark, males compensate for the missing vision by changing their courtship strategy from visual tracking to scanning (M5). This scanning behavior is triggered by the decamping female (M2) and driven by volatile (M3) and contact pheromones (M4). Even though gustatory and olfactory signals show significant overlap in sustaining courtship, they are probably both necessary under natural conditions to enable the male to respond quickly to the presence of a potential mate. Finally, feedback behavior of the female, probably genital and wing spreading [3], is necessary for copulation to occur (M13).

It should be emphasized that, although both olfactory and gustatory signals trigger initiation of courtship in the dark, they are not sufficient to stimulate the courter in the absence of a courtee [26]. This implies the presence of additional stimuli that are necessary but, as we have demonstrated, also not sufficient to trigger courtship initiation in the dark. Rather these stimuli need to be integrated with chemosensory signals to arouse the male. Since these signals can originate from decapitated objects, as shown here, or from “fly-like dummies” [26], we propose, in agreement with others [25], [26], that decapitated object flies are a source of tactile cues to the courting male (indicated as “tc” in the model of Figure 9).

Remarkably, if the courtee is decapitated, courtship initiation of a sexually naïve male is not exclusively directed towards females (M14, M18, M27), but males seem to be almost as attractive (M15, M19, M28). After initiation of courtship in the dark, however, males with intact gustatory modality show a clear preference towards decapitated females (M16, M17). Although gustatory signals carry sex-specific information, they provide positive stimuli when received from both decapitated females (M16) and decapitated males (M17). Interestingly, in the dark, lack of taste perception does not lead to a change in male–male courtship intensity. Rather it is the integration of repellent feedback signals (M22) with the sex-specific gustatory signals (M25) that suppresses male–male courtship in the dark. Accordingly, we propose that the tapping step in the courtship ritual [3] is not only used to obtain gustatory information from the potential mate but also to provoke a response.

Although visual information plays a prominent role in the sexual orientation of males (M26, M29), homosexual courtship is fully suppressed only in combination with gustatory and feedback signals (M25, M30, M31). This integration of sensory information might be especially important for a male's sexual orientation in a natural situation. Although it is unclear which senses are stimulated by the feedback signals, it is obvious from our results that males do take advantage of these signals to distinguish between males and females (M22). Indeed, as taste-deficient males scan for dewinged, but not intact, males in the dark, auditory cues generated by wing scissoring may play a role as inhibitory feedback signals. Finally, olfactory cues are neither sufficient nor necessary to inhibit male–male courtship, but elicit attractive stimuli when originating from females as well as males (M20, M21).

### Light-dependent behavioral switch

A male adopts different strategies to pursue a decamping female, depending on the presence of light. While he tracks the female in daylight, he searches her in the dark by scanning the courtship chamber. Interestingly, in daylight lack of visual acuity is not sufficient for blinded males (black-eyed or *ninaB^360d^*) to adopt the scanning behavior. Therefore, it is the perception of light rather than visual acuity that controls the choice between these alternative strategies. Blind males could perceive light that regulates this switch in various ways. (i) The photoreceptors of black-eyed males might be activated in the ocelli or by light penetrating the head cuticle. (ii) As the electroretinogram of *ninaB^360d^* males is altered but still shows a response to light [43], retinal-independent photoreceptors or unknown light-gated channels might be the light-sensitive receptors regulating the switch. (iii) Since males display the scanning behavior under red light during the circadian day, we assume that it is independent of the circadian clock. Nevertheless, extraretinal photoreceptors in the CNS, for example those adjusting the circadian clock in the brain (cryptochromes) [52], [53] might control the scanning behavior of males. As it was previously reported that visual mutants (*e^11^*, *tan^1^*) zigzag in daylight [30] and we observed a similar effect with *norpA^P24^* mutant flies [54] (data not shown), functional dissection of these genes may shed light on the neuronal and molecular control of this light-induced behavioral switch.

Quantification of the scanning behavior, which might be desirable, is met by two main difficulties that require a more elaborate setup. During the recording with an infrared camera flies move out of focus in a courtship chamber of 9 mm height, which cannot be reduced significantly without seriously affecting the courtship behavior (see below). Moreover, males also scan along the walls of the chamber (Movie S1). Hence a more sophisticated setup is required that follows and records the moving males in focus to permit integration of these data for further analysis. However, it should be stressed that for our model a quantitative analysis of the scanning behavior is irrelevant because the model only incorporates our observations that (even blind) males never scan in daylight, while they always scan in the dark, independent of their genotype with the exception of *Poxn-pRes*; *Or83b* ^2^ males that virtually do not court in the dark (Figure 3C).

### Initiation of courtship: dual role of female movements

It has been observed that males exhibit a high CI only if the female is able to move [32]. By contrast, we observed a high cvi also towards immobile decapitated females. In this context it is instructive to compare, in addition to the courtship vigor, courtship initiation of wild-type males towards intact and decapitated females. Although in daylight all *Ore-R* males initiated courtship towards intact (Figure 3A) and decapitated females (Figure 6B), intact females appear more attractive to them than decapitated females, as the latency till courtship initiation doubles when females are decapitated (cf. *Ore-R* in daylight in Figure 3B and Figure 6C; p = 0.048). It follows that, in contrast to a decapitated and hence immobile female, a moving female enhances the male's arousal and thus reduces the latency till courtship initiation. Since computation of the CI, yet not of the cvi, includes the latency and thus reduces the CI in comparison to the cvi, it is plausible that the low CI towards immobile females [32] results from the prolonged latency rather than a reduction of the courtship vigor.

A result opposite to that in daylight, however, is observed in the dark, where the latency till courtship initiation is reduced with decapitated as compared to intact females (cf. *Ore-R* in the dark of Figure 4B and Figure 3B; p = 0.005). In the dark the movements of a female obviously lose their attraction, as the male cannot see them. Instead, an intact female decamps when the male bumps into her, whereas a decapitated female remains immotile. Thus, while the male begins to court by extending his wing only after several brief encounters with an intact virgin female in the dark, a decapitated female is a stationary source of attraction and induces a male to court upon their first contact. It is possible that the male interprets the non-escaping behavior of the decapitated female in the dark as acceptance behavior. Alternatively, the extended duration of contact with her may lead to a stronger chemosensory stimulation. This effect with decapitated animals also depends on the integration of gustatory with olfactory pheromonal signals, as shown here and by others [55]. If olfactory or gustatory cues are not perceived by the male, latency till courtship initiation is considerable prolonged in the dark (compare *Ore-R* with *Or83b^2^* or *Poxn-pRes* in Figure 4B).

### Chemosensory signals and sexual orientation

Our conclusion that gustatory cues carry sex-specific information is consistent with the notion that sexual orientation in *Drosophila melanogaster* depends on cuticular hydrocarbons (CHs), the predominant forms of which display a sexual dimorphism with high levels of 7,11-dienes on females and of 7-tricosene on males [56]. Moreover, drastic reduction of unsaturated CHs in object animals reduces the CI of courting males as well as their ability to recognize the sex of the courtee [21]. Our conclusion is further in agreement with the recent demonstration that mutants in the gustatory receptor Gr32a court decapitated males in daylight with an enhanced CI [24].

Contrary to a recent report, which claims that males without the olfactory pheromone receptor, Or67d, for 11-*cis*-vaccenyl acetate (cVA) inappropriately court intact males [28], we find that olfactory cues are neither sufficient nor necessary to inhibit male–male courtship (Figure 9). In an attempt to resolve this inconsistency, we examined under our assay conditions the courtship behavior of *Or67d^Gal4^* mutants, in which the open reading frame of the *Or67d* gene has been replaced by that of *Gal4* [28]. In contrast to the published results [28], we found that *Or67d^Gal4^* males, like *Ore-R* males, do not court intact males significantly in daylight (Figure S2A). In the dark, the CI of *Or67d^Gal4^*, as compared to that of *Ore-R*, males is slightly but significantly elevated (Figure S2C; p = 0.036), in apparent agreement with the published results [28]. However, when we tried to rescue the *Or67d^Gal4^* males by expressing the Or67d receptor under the control of Gal4, their CI remained indistinguishably elevated (Figure S2C; p = 0.96). Therefore, we must attribute this small effect on male–male courtship in the dark to the *Or67d^Gal4^* chromosome. The fact that the CI of *Or67d^Gal4^* males is not significantly increased in daylight (Figure S2A) is explained by our finding that vision plays an important role in the suppression of male–male courtship (Figure 7). We have further examined the courtship of *Or67d^Gal4^* males with decapitated males in daylight and in the dark. These results confirm our observations with intact object males (Figure S2A,D), as explained in the legend to this figure.

The obvious question is why do our results on *Or67d^Gal4^* males courting wild-type males in daylight differ so drastically from those previously reported [28]. While there might be several small differences between our experimental setups, the most obvious is the difference in height and diameter between our courtship chamber (9 mm×16 mm) and that used previously (4 mm×10 mm) [28]. That the dimensions of the chamber are important becomes evident when copulation efficiencies are measured. Under our conditions, wild-type flies copulate with an efficiency of 100% within 4–5 minutes (Figure S2B). By contrast, only about 60% of wild-type flies copulated within 30 minutes in the smaller courtship chamber [28]. Thus, too small a chamber may stress flies and influence their courtship behavior drastically. We therefore conclude that the earlier results on male–male courtship [28] are affected by suboptimal conditions of the courtship assay and hence may mislead others [57], [58]. However, we do not question a repellent role in courtship of cVA, a pheromone detected by the Or67d and Or65d receptors [28], [59]. This pheromone, synthesized in the male accessory gland and transferred with the sperm to the female [60], [61], renders the mated female less attractive for males [62]. In fact, we have observed that olfactory-deficient *Or83b^2^* as compared to wild-type males increase their courtship vigor towards mated females that had been decapitated and hence displayed no rejective behavior (data not shown).

### The complexity of male chaining behavior

Males courted each other and formed courtship chains when deficient for gustatory perception but not when deficient only for olfaction (Figure 8). Why did these males chain even though we expect, from single-choice courtship assays, visual and negative feedback signals to inhibit male–male courtship? A plausible answer is that, in this crowded and more complex situation, males may have to deal with additional stimulatory and reduced repellent cues. It is known, for example, that a male's love song arouses, and enhances the locomotor activity of, other males [63], [64]. It is also possible that the rejective behavior of a courted male is reduced when he tries to court another fly at the same time. Therefore, it is conceivable that the loss of gustation, but not of olfaction, disturbs the fine-tuned balance of stimulatory and inhibitory signals and affects the male's ability to properly discriminate against other males. Integration of these gustatory signals with visual (M30) and behavioral (feedback) cues (M25) might be especially important for the male's sexual orientation in a natural situation on a patch of food attracting many flies of both sexes. Finally, it is difficult to correctly interpret results obtained in this complex chaining assay because the courting as well as the courted males share the same sensory defects, which probably not only affects the behavior of the courters but also that of the courtees.

### Logic of neural network regulating male courtship behavior

Our model (Figure 9) describes the logic of the program that controls the courtship behavior and sexual orientation of the *Drosophila* male by integrating the various sensory inputs and converting them into a complex behavioral response. This program is executed by the underlying neural network, which is specified, as the behavior is innate, through the genetic program during development. In principle, it is the intrinsic properties of the neurons and their wiring that determine the program regulating this behavior. Our model will provide crucial information for understanding the intrinsic properties of the neural network, once its wiring has been established. This information is important because we are convinced that it is impossible to understand how a neural network controls behavior, simply on the basis of knowing its architecture [18]. Although one might suspect that knowledge of the wiring of the neural circuitry as well as of the intrinsic properties of all neurons participating in it would be sufficient to understand its properties as a regulatory circuit, the task to acquire this knowledge is exceedingly difficult. The processing of the sensory signals whose logic our model explains is expected to be reflected by a homologous neural network of the male fly. Accordingly, our model illustrates the logic of the neural circuit that regulates male courtship behavior and thus will complement studies that determine the wiring and intrinsic properties of the neurons forming this circuit.

While our model attempts to provide the most complete picture of male courtship control by sensory stimuli, it is also limited by the requirement for standardized conditions to obtain reproducible results. These restrict the model to the simple paradigm of single-choice courtship behavior. In addition, isolation of males and virgins after eclosure for 4 to 5 days before they are united in single-choice courtship assays guarantees that the observed behavior is innate and initiation of courtship is not delayed by what appear erratically extended periods. For these reasons, we have avoided to indicate quantitative features in our model and have restricted the model to only reflect the logic by which sensory stimuli influence male courtship behavior. It is probable that courtship studies under more natural and complex situations than those used here will provide a much more detailed picture of male courtship behavior and hence necessitate modification of the model. Such more natural situations may further have to take into account that there might be a spontaneous courtship behavior of males independent of external stimuli, which does not occur in our simple paradigm but has been observed for the turning behavior of tethered flies [65]. Although future experiments will modify and extend our model, we are confident that the general features of the model and its logic will stand.

## Materials and Methods

### Fly stocks

The genotype of the transgenic *Poxn-pRes* flies is *ΔXBs6*; *Poxn^ΔM22-B5^/Poxn^ΔM22-B5^ ΔPBs96.2*, that of the transgenic *Poxn-SuperA* flies is *Poxn^ΔM22-B5^ SuperA-158* (cf. Figure 1). *Or83b^2^*, *ninaB^360d^*, and *Or67d^Gal4^* mutants were kindly provided by Leslie Vosshall, William Pak, and Barry Dickson.

The genetic background of the stocks used in this study could not be strictly controlled due to the long period of this study. The *ninaB^360d^* stock [43] was out-crossed four times with *w^1118^* flies prior to the experiments. *Or83b^2^* flies [36], *Poxn^ΔM22-B5^* mutants, and the flies carrying the *Poxn* transgenes [42] were also in a *w^1118^* background, but not out-crossed. Finally, before their use in courtship assays, the X-chromosome carrying the *w^1118^* mutation was exchanged for the X-chromosome of *Ore-R* flies in all stocks. Since all behavioral phenotypes of *Poxn-pRes* flies were rescued by the *SuperA* transgene of *Poxn*, we could exclude that these phenotypes resulted from the genetic background rather than the mutated *Poxn* gene. Although we cannot rule out that the *Or83b^2^* stock has accumulated modifiers over time, our behavioral analysis showed that the *Or83b^2^* mutation suppresses olfactory perception important for courtship.

### Courtship assay

Flies were cultured and single-choice courtship assays performed essentially as described [42]. We would like to emphasize that the size and shape of the courtship chamber are critical to observe the decamping of the female and subsequent searching behavior of the male (i.e., visual tracking versus scanning). Our chamber (9 mm height×16 mm diameter) was prepared from a 24-well plate (Cellstar, Greiner Bio-One) by cutting off its top. It fulfills our criterion that *Ore-R* flies copulate at 100% efficiency within 5 minutes in single-choice assays in daylight. Courtship was observed during 0–3 hrs and 8–12 hrs circadian time (CT; light is on from 0 to 12 hr CT) when flies are most active [47], except for flies carrying the *ninaB^360d^* mutation, which were observed during 1–4 hrs CT, as courtship was significantly reduced in evenings (8–12 hrs CT). Observation in the dark was performed 10 minutes after moving the flies from the light to dim red light during the circadian times mentioned. The courtship vigor index, cvi, is defined as fraction of time the male spent courting from courtship initiation until copulation or the end of observation at 10 minutes, whereby any of the following behaviors were scored as courting [3]: wing vibration, tapping, licking, bending the abdomen, orienting, following with extended wings, and scanning. By contrast, the courtship index CI is defined as the fraction of time the male spent courting from the beginning of observation until copulation or the end of observation at 10 minutes. Thus, the CI does not distinguish between courtship initiation and maintenance because it includes the latency interval as well as all males that do not initiate courtship during the observation period. Copulation efficiency was defined as the number of males copulating divided by that initiating courtship within the 10 minutes of observation.

### Chaining assay

Chaining assays were performed with eight sexually naïve males in a 10 mm×35 mm Petri dish filled with a 6 mm agar layer. Chaining indices, defined as the percentage of time three or more males form a chain during a 10 minutes observation period, were calculated essentially as described [66]. However, the behavior of the males was assayed immediately rather than a day after the males had been placed into the Petri dish [13].

### Non-genetic manipulations of subject and object flies

For some assays the eyesight of subject males was blocked by black nail polish one day before the experiment. Object animals were decapitated and kept in a humid environment for an hour before the assay. Only decapitated flies that did not react to mechanical stimuli, but showed grooming behavior, were selected for tests. Object males were dewinged by clipping their wings immediately distal to the hinge one day before courtship assays. Intact object males were marked by slightly clipping the distal wing edges one day prior to the experiments. All these manipulations were performed on flies anesthetized by CO_2_.

### Statistical methods

Where applicable, results were tested for following a normal distribution by the ‘Shapiro-Wilk test’ [67]. While 86% of the data sets obeyed a normal distribution (p<0.05), the remaining 14% of data sets, all of which consisted of a small number of observations (n∼10), may also follow a normal distribution with a Wilk number between 0.7 and 0.8, although their p-values are larger than 0.05. Hence, all mean values were compared on the basis of the two-tailed Student's t-tests. The significance of (i) differences between fractions of males initiating courtship, and (ii) fractions of males initiating copulation was computed by Pearson's χ^2^-test with Yates' correction. To avoid overloading, we omitted significance markers from the figures. Instead we indicated the p values in the text and used double standard error bars to facilitate “comparison by eye” [68].

## Supporting Information

Figure S1 Courtship indices (CIs) for courtship assays shown in Figures 2 to [Fig pone-0004457-g003] [Fig pone-0004457-g004] [Fig pone-0004457-g005] [Fig pone-0004457-g006] 7.
(0.39 MB PDF)

Figure S2 Sexual orientation of *Or67dGal4* mutants.
(0.40 MB PDF)

Movie S1 Two courtship assay clips demonstrating the behavioral strategy of an *Ore-R* male retrieving a decamping *Ore-R* female in the dark (first part) or in daylight (second part). In the dark, the male is scanning the courtship chamber in search for the female. His wings are slightly spread and he moves in a zigzag pattern. In daylight, the male uses his visual capabilities to orient towards and follow a decamping female.
(0.77 MB MOV)

Movie S2 Courtship assay clip of a blind (*ninaB360d*) *Drosophila* male in daylight. The male does not follow a decamping *Ore-R* virgin female nor switch to the scanning behavior. Although the male is unable to orient towards the female using visual cues, it appears to do so when the female is very close, using its other senses. Trying to retrieve the female, the male often displays short vibrations with one or both of his wings. This behavior was considered as courtship behavior and included in the computation of the cvi accordingly.
(0.55 MB MOV)

Movie S3 Courtship assay clips of a taste-deficient *Poxn-pRes* male (marked by circle) courting (i) an intact (first part) or (ii) dewinged (second part) *Ore-R* male in the dark. In the first clip, it is obvious that the *Poxn-pRes* male continues to court and even attempts to mount despite negative feedback cues (e.g., wing flicking) from the wild-type male. The second clip shows the scanning behavior of the *Poxn-pRes* male after losing contact with the dewinged male. This behavior, which is similar to that displayed during heterosexual courtship, is rare.
(7.38 MB MOV)

Movie S4 A chaining assay clip showing eight sexually naïve *Poxn-pRes; Or83b2* males in a small Petri dish (Materials and Methods). Four of the males are forming a courtship chain. Note that this clip was taken 10 minutes after the males have been placed into the Petri dish.
(0.34 MB MOV)
