# Supplementary material for: Sensory Integration Regulating Male Courtship Behavior in Drosophila
Source: PLoS One. 2009 Feb 13;4(2):e4457. doi: 10.1371/journal.pone.0004457 (PMC2636894; doi:10.1371/journal.pone.0004457)
Supplement: Figure S1 — Courtship indices (CIs) for courtship assays shown in Figures 2 to 7. (0.39 MB PDF) [file pone.0004457.s001.pdf]

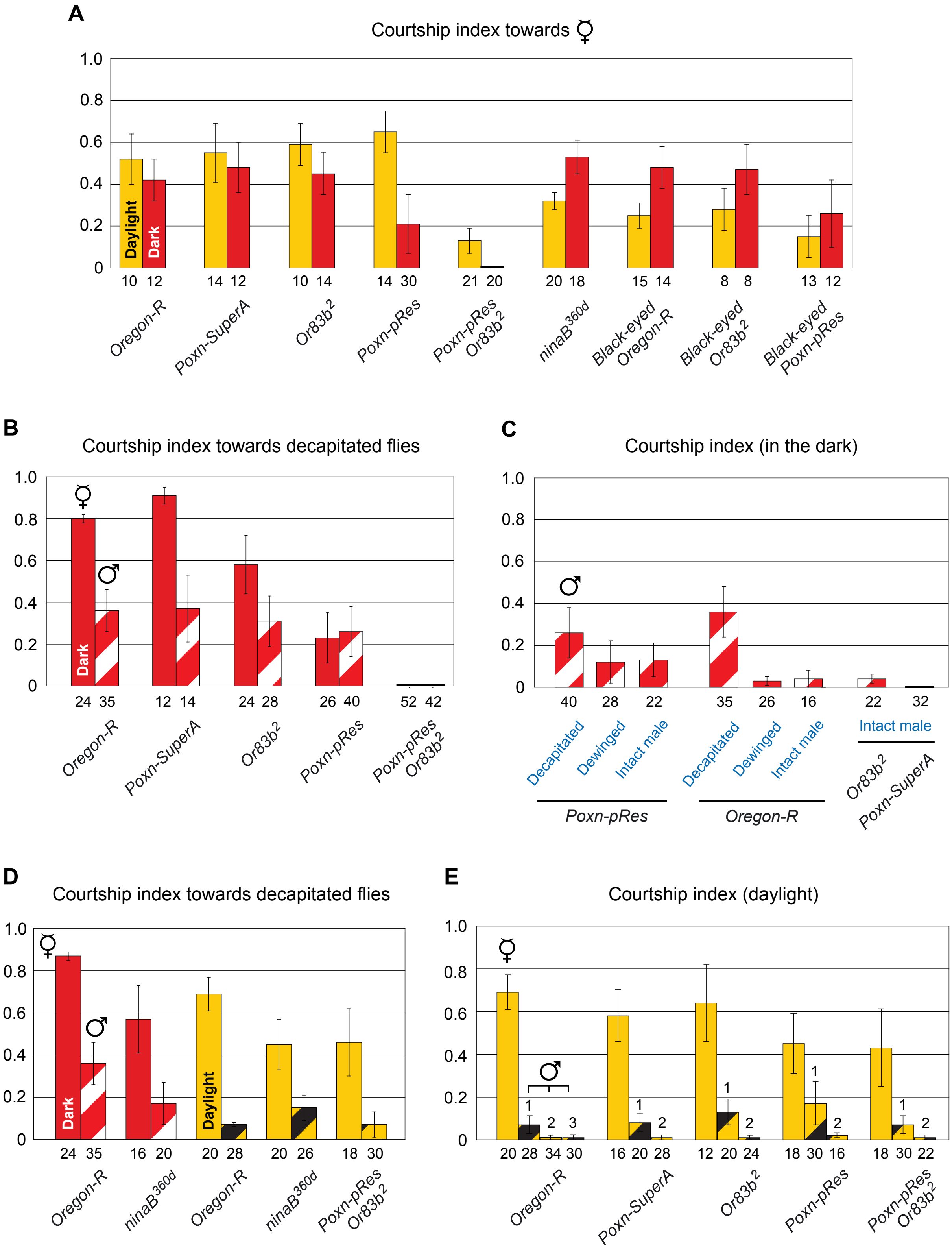

Figure S1 Courtship indices (CIs) for courtship assays shown in Figures 2 to 7. CIs were computed for courtship assays of Figures 2 and 3 (A), Figure 4 (B), Figure 5 (C), Figure 6 (D), and Figure 7 (E). For explanation, see legends to these figures.
