# Supplementary material for: Sensory Integration Regulating Male Courtship Behavior in Drosophila
Source: PLoS One. 2009 Feb 13;4(2):e4457. doi: 10.1371/journal.pone.0004457 (PMC2636894; doi:10.1371/journal.pone.0004457)
Supplement: Figure S2 — Sexual orientation of Or67dGal4 mutants. (0.40 MB PDF) [file pone.0004457.s002.pdf]

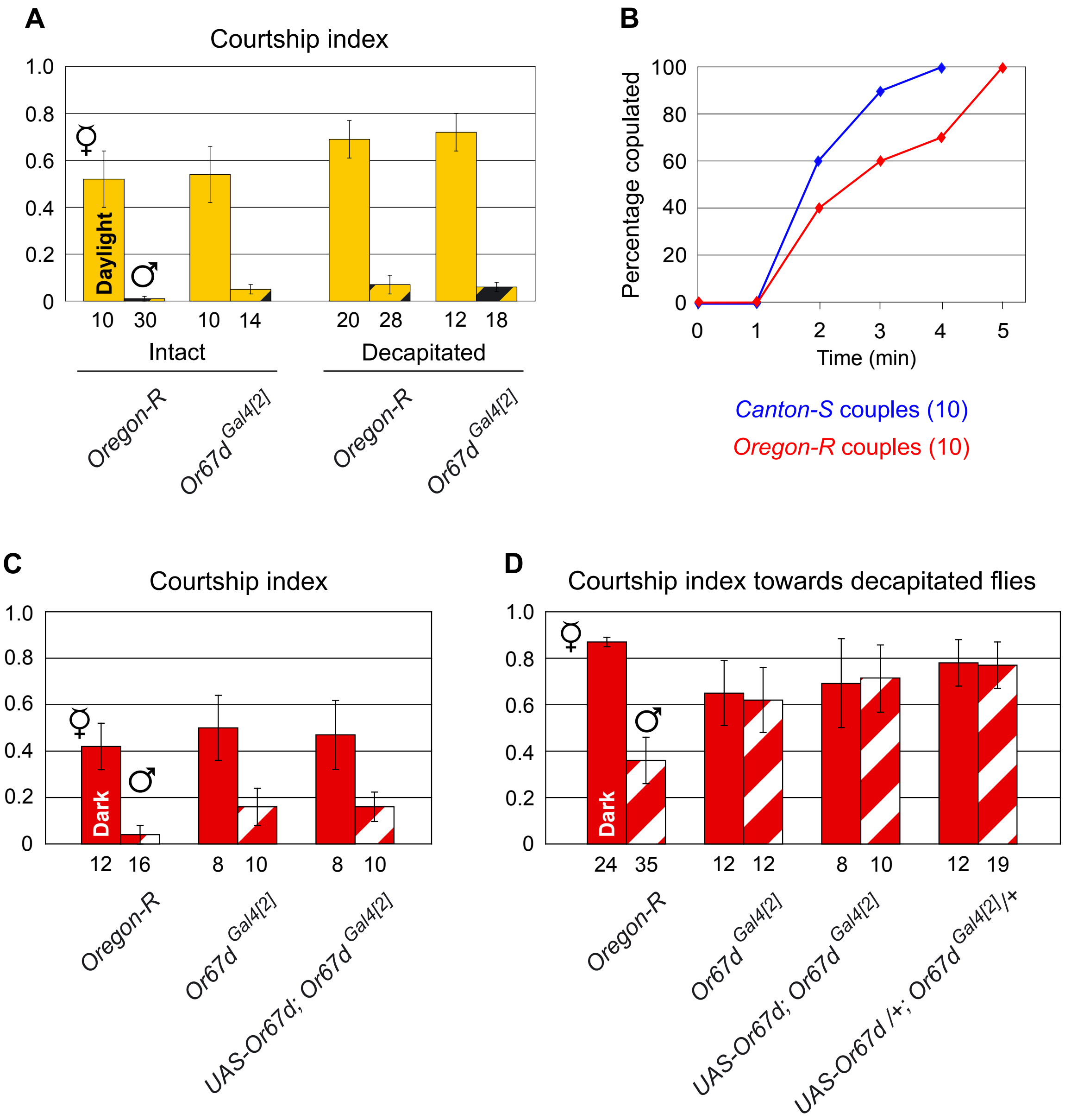

Figure S2 Sexual orientation of $Or67d^{Gal4}$ mutants. CI values were measured in single-choice courtship assays with mature males of indicated genotypes and (A) intact or decapitated *Ore-R* virgins (♀, filled columns) and males (♂, hatched columns) in daylight, (C) intact *Ore-R* virgins (♀, filled columns) and dewinged males (♂, hatched columns) in the dark, and (D) decapitated *Ore-R* virgins (♀, filled columns) and males (♂, hatched columns) in the dark. The number of couples observed is shown below each column. Error bars represent double s.e.m. (B) Copulation efficiencies of wild-type couples in single-choice courtship assays in daylight. The percentage of copulating *Canton-S* (blue graph) and *Ore-R* couples (red graph) out of 10 couples each is plotted as a function of time they spent in a cylindrical chamber of 9 mm height x 16 mm diameter.

In addition to single-choice courtship assays with intact flies, we conducted experiments with virgins and males that had been decapitated (A,D). In daylight, $Or67d^{Gal4}$ compared to *Ore-R* males did not increase their CI towards decapitated males (p=0.11), as observed with intact object flies (A). By contrast, in the dark (D) the CI towards decapitated males of $Or67d^{Gal4}$ compared to wild-type males was increased significantly (p=0.001) and was the same as that towards decapitated females (p=0.77). However, this phenotype of $Or67d^{Gal4}$ males could not be rescued by expressing Or67d under the control of Gal4 (D). Since the CI towards decapitated males of heterozygous *UAS-Or67d*/+; $Or67d^{Gal4}$/+ males was increased as well, we conclude that the $Or67d^{Gal4}$ insertion or the genetic background of this fly stock generates this dominant courtship phenotype.

It should be emphasized that the $Or67d^{Gal4}$ stocks, kindly provided by Barry Dickson, were verified for the replacement of the open reading frame of *Or67d* by that of *Gal4* [28] by isolation of their DNA, followed by PCR and DNA sequencing of the insertion site.
